# The Medical Professional Elimination Program and the Ideology and Motivation of Nazi Physicians

**DOI:** 10.5041/RMMJ.10533

**Published:** 2024-10-28

**Authors:** George M. Weisz, Deborah E.-S. Hemstreet

**Affiliations:** 1 School of Humanities, University of New South Wales, Sydney, Australia; 2 School of Humanities, University of New England, Armidale, Australia; 3 English Communications Coordinator, Rambam Health Care Campus, Haifa, Israel; 4 Editorial Assistant, Rambam Maimonides Medical Journal, Haifa, Israel

**Keywords:** Böhler, Eberl, Heissmeyer, medical ethics, Nazification of medicine, Sauerbruch

## Abstract

The appointment of a new chancellor in 1933 marked the beginning of the Third Reich in Germany. The ideology of the Nazi Party focused on establishing a pure Aryan state characterized by nationalism and racial superiority. Their goals would be achieved through a totalitarian form of government that enforced the subjugation, exclusion, and elimination of those they defined as inferior minorities, particularly Jews, who were depicted as non-human. Implementation of the Nazi ideology required the exclusion of Jewish people and other dissenters, particularly Jewish physicians, from their professions. The exclusion of Jewish physicians, referred to herein as a “Medical Professional Elimination Program,” was gradually imposed on other Jewish professions in nations absorbed by the Third Reich, and particularly enforced by incorporated Austria. Why did German and Austrian doctors support the Nazi racial ideology, the removal of Jewish physicians from every possible sphere of influence, and subsequently participate in criminal medical research and experimentation, as well as euthanasia of perceived non-contributors to society, and become involved in refining the effectiveness of the death camps? Was the Medical Professional Elimination Program an opportunistic political concept, or was it part of an entrenched ideology? With these questions in mind, the lives of four key Nazi physicians and two institutions are examined.

## INTRODUCTION

Since the end of World War II, the medical practice abuses performed by Nazi physicians under the Third Reich have been hotly discussed and debated. Elie Wiesel, a prolific author and Holocaust survivor, wrote: “During the period of the past century that I call NIGHT, medicine was practiced in certain places not to heal but to harm, not to fight off death but to serve it.”^1^^(p1511)^

Robert J. Lifton points out that a primary goal of the Third Reich—to have a pure Aryan population—would not have been possible without the step-by-step removal of the Jewish people from all spheres of public influence.^2^ While anti-Semitism was strong in pre-Nazi Germany, with the election of a new leader, laws of subsequent severity were passed that made anti-Semitism ethical based on the ideology of racial (Aryan) superiority. The first such law was enacted on April 4, 1933: The Law for the Restoration of the Professional Civil Service,^3^ commonly referred to as the *Berufsbeamtengesetz*, meaning “Professional Civil Service Act.” The title of the law would have appealed to a nation seeking positive change, presenting an assumption by the general public that its implementation would lead to a more efficient national and professional civil service. In reality, this law mandated the dismissal of any tenured civil servants who were not sufficiently Aryan or with questionable political affiliations. It was followed by the 1935 Nuremberg laws which imposed additional restrictions, such as prohibiting Jewish doctors from treating non-Jewish patients. In July 1938, the medical licenses of all Jewish doctors were rescinded. Furthermore, following the *Anschluss* (Germany’s incorporation of Austria) in 1938, the above laws were enthusiastically enforced in Austria, particularly among Austria’s medical professionals.

The widespread support of these laws raises multiple questions. The rapidity with which the Act and subsequent laws were enforced, particularly on Jewish medical practitioners, effectively removed them from all academic positions, medical institutions, and private practice. Hence, hereinafter, the term “Medical Professional Elimination Program” will be used when referring collectively to these laws in relation to their impact on the Jewish medical profession.

What inspired the support of so many German medical professionals throughout the Holocaust? Was it their political or scientific ideology, opportunism, career advancement, or the potential for financial gain?^4^,^5^ No laws were passed requiring the participation or cooperation of physicians with the Nazi policies or programs.^4^,^6^ Yet, approximately 45% of the medical community in Germany became Nazi Party members, in contrast to only 1% of the general population, and 6% were already involved in the Nazi Party before its rise to power. Furthermore, 7% of medical professionals enrolled in the violent SS (*Schutzstaffel* or “Protective squadron”), compared to 1% enrolled from within the general population.^5^,^7^–^9^ Additionally, compared to the Germans, the Austrians were notably more efficient and faster in implementing the Medical Professional Elimination Program.^10^

With that historical background in mind, motivations will be considered for their support of the Medical Professional Elimination Program and subsequent activities that undermined their commitment to humanity as physicians. The lives and possible motivations of four Nazi physicians (two German and two Austrian) who actively or passively supported the Program are examined and discussed, together with a glimpse of the institutions that may have also been involved.

## HISTORICAL BACKGROUND

The Jews of Germany and Austria were no strangers to anti-Semitism. Extensive histories of German and Austrian Jewry and anti-Semitism are available elsewhere.^11^–^13^ Despite the presence of anti-Semitism, the Jewish communities in these nations thrived, particularly after Austria (in 1867) and Germany (in 1871) passed legislation providing equal rights and citizenship to their Jewish residents. The liberated Jews proved themselves in academia, particularly in science and medicine.

In the pre-Nazi decades, German universities were the most sought after in the world. German Medical Scientists were recognized worldwide and had received the highest numbers of Nobel recognitions between 1901 and 1936 (41 awardees). Interestingly, close to 25% of the German Nobel laureates were Jewish.^14^ By 1933, although only 4% of Berlin’s population was Jewish, Jewish physicians occupied nearly 40% of all medical positions.^9^

Austria, on the other hand, was a major contributor to the international literature, most of which was published in German.^10^ Prior to the *Anschluss* in 1938, there was a thriving Jewish population (~192,000), most of whom lived in Vienna. In 1910, half of the Medical Faculty of Vienna were of Jewish ancestry. Although Vienna had a 2% Jewish population, Jews held the majority of all the city’s medical positions (3,200 out of 4,900 practicing physicians in Vienna).^10^,^15^–^17^ This was most likely because, historically, medicine was the only academic profession open to Jewish people.^10^

Despite religious and societal differences, the Jewish communities of Germany and Austria had seemed to successfully integrate with their respective societies. But the historic roots of anti-Semitism, when mixed with the political and economic fall-out of World War I and social Darwinism, would become fodder for the Nazi ideology. The lives of German and Austrian Jewry, who truly loved and served their respective nations, would soon be irrevocably changed.

### Germany and Austria: Post World War I

Both Germany and the Austro-Hungarian empire were severely affected by their defeat in World War I. The terms of the Treaty of Versailles for Germany^18^ and the Treaty of Saint-Germain-en-Laye for Austria^19^ were devastating. Offering a vision for national revival, stability, and prosperity, along with Aryan superiority, the fledgling Nazi Party gradually grew stronger, despite intense opposition from conservative and other political parties.^20^ With World War I lost, the peoples of both nations looked for and found a scapegoat. Walter Rathenau, a Jew, was Germany’s foreign minister from January 1922 until his assassination in June. He was instrumental in the country’s observation of the terms of the treaty. However, right-wing groups, which included the fledgling Nazi Party, saw him as a Jew making deals with other Jews.^21^ Rathenau was one of many who would be blamed for the post-war suffering of Germany and Austria—anti-Semitism again increased.

Interestingly, anti-Semitism was initially more overt in Austria than in Germany. By February 1934, even before the *Anschluss* in March 1938, no Jews were being appointed or promoted in Viennese hospitals or welfare institutions.^10^ Medical, scientific, and academic appointments were made based on both religious and racial considerations, and no non-Aryans (i.e. Jews) were appointed. In 1937, doctors seeking employment in public hospitals were required to present baptismal certificates. Jewish patients were not treated unless they had private medical coverage, and only in the remaining Jewish hospital (Rothschild) which was closed by the Nazis in 1943.^22^

### Eugenics and Social Darwinism: Impact on German and Austrian Medicine

In pondering the actions of Nazi doctors, Nobel laureate and Auschwitz survivor Elie Wiesel asks: “What made them forget or eclipse the Hippocratic Oath? … What happened to their Humanity?”^1^^(p1511)^

Few are aware that the idea of Aryan superiority had already been proposed by a French aristocrat and anthropologist, Count Joseph Arthur de Gobineau, in 1853. In 1859 Darwin published his seminal *On the Origin of Species*. Darwin’s theories particularly caught hold in Europe. The term “eugenics” was subsequently defined in 1883 by an Englishman (Francis Galton), and a German (Alfred Plontz) first wrote about racial hygiene in 1895.^9^ Combined, these concepts converged to a unique development of Germanic social Darwinism—which sought to apply the same laws of natural selection observed in nature to human groups and races.^23^,^24^

Aryan superiority, social Darwinism, and eugenics were not simply ideas posed for discussion. They were actualized in the genocide of the Hehero and Nama peoples in the German colony of Namibia.^25^ Indeed, what occurred in Namibia was a final solution process hauntingly similar to that of the Nazis.

Hence, the answer to Wiesel’s question rests in a complex interweaving of the German “empire” (*Reich*)-centric and racist ideology with the impact of Germanic social Darwinism on German and Austrian medical professionals—well before the Nazis’ rise to power. Fearing the degeneration of the Aryan genetic pool, German social Darwinists were already developing a theory of eugenics at the end of the 19th century.^2^ The result was an intermingling of medicine with political affiliation, driven by the Nazi ideology.^3^^(p408–9;422–5)^

Of particular relevance to the topic of this paper was establishment of the National Socialist Physician’s League, aimed at coordinating Nazi medical policy and removing all Jews and communists from the sphere of medical influence. The organization represented 6% of the medical profession, and by January 1933, before the Nazis came into power, 38,000 physicians had joined the Nazi Party. By 1942, nearly half of all doctors in Germany belonged to the Party and the League. Proctor saliently points out: “medical scientists were the ones who invented racial hygiene in the first place.”^3^^(p409)^

The ideology of racial hygiene also caught on in Austria and likewise became intertwined with politics. Establishment of the Society of German Doctors in Austria led to the publication of lists of non-Aryan colleagues. This climate of increasing anti-Semitism led to the emigration of most Jewish colleagues working in hospitals and academia before 1938.^10^

Ultimately, the Hippocratic oath was redefined.^26^,^27^ Social Darwinism and so-called “scientific” racial hygiene would become part of the complex groundwork that led to euthanasia, the death camps, and medical experimentation on non-Aryan subjects.^28^

## THE MEDICAL PROFESSIONAL ELIMINATION PROGRAM

The turning point for German Jewry, and by extension all who would come under Nazi rule, began on April 7, 1933, when the Professional Civil Service Act became law. As a law, it marked the official beginning of what is referred to herein as the Medical Professional Elimination Program.

The Program had a particularly strong impact on German doctors. In January of 1933 there was a surplus of German physicians, and the ladder for advancement was long, with poor remuneration.^9^ By early 1934, 2,600 Jewish physicians had been dismissed from their positions, all of which were rapidly filled by Aryans. The marked reduction in the number of available physicians, thanks to the removal of the Jews, meant increased salaries and professional advancement opportunities for the German physicians. Furthermore, perhaps because of the importance of physicians for the advancement of the Nazi ideology, doctors became among the best paid professionals in the Third Reich.^9^

In 1938, the *Anschluss* was welcomed by the majority of Austrians. Doctors made up the largest proportion of Austrian Nazi Party membership, and, as mentioned above, many of their Jewish colleagues had already been expelled from their positions.^15^ With the *Anschluss*, some 3,000 doctors were immediately removed from the *Ärztekammer* (Medical Chamber), 65% of whom were Jewish. In parallel, 372 Jewish doctors received the status of *Krankenbehandler (*literally, patient handler, i.e. medical attendant) and could treat only Jewish patients; 153 doctors lost their medical faculty positions as professors and students.^10^

Dismissal of the Jewish physicians and professors had tragic consequences. Many Jewish doctors committed suicide.^29^ Most escaped to Great Britain, some escaped to the United States and even Palestina (formal name in pre-Israel Hebrew), while others found themselves traveling from country to country in Europe and the Far East as the Third Reich expanded.^10^,^15^,^16^

### The End-Goal

The Medical Professional Elimination Program was indeed well planned. The highly recognized psychiatrist and author, Robert J. Lifton, points out that the limitations on Jewish medical practice in early 1933 were but the beginning of a step-by-step process to end *all* Jewish medical practice, since doing so all at once would have devastated German medical practice.^2^ Hence, the German and Austrian doctors benefited at multiple levels: they had an unhindered pursuit of racial hygiene, of which anti-Semitism played a major role, and could realize the professional and economic benefits that the Jews had received as the major players in medicine.^2^

The Medical Professional Elimination Program facilitated the Third Reich’s ideology of achieving Aryan racial purity at any cost. What followed could not have been achieved without the support and aid of the Aryan medical profession.^2^,^3^,^28^ The Program started out as a simple “rehabilitation” of the professional civil services, but that Act paved the way for what would follow, all carefully legislated, law upon law.^3^ Among these laws were: The Sterilization Law of July 1933, minimizing the reproduction of “defectives” in 1934, and the Nuremberg Laws of 1935 aimed at promoting racial purity, to name a few. The introduction of euthanasia programs in 1939 facilitated the rapid implementation, in 1941, of the mass murder of the Jews and other non-Aryans, medical experimentation, and more.

The first official instance of euthanasia in Germany occurred in 1939 at the Leipzig Children’s Hospital.^4^,^28^ That it was performed on a so-called “monster” at the request of his parents is already shocking. But what followed after the child was euthanized would have far-reaching consequences. Initially, the hospital physicians refused to kill the child without receiving permission from the Führer. The Head of State dispatched his private physician, Karl Brandt, to confirm the need to euthanize this specific newborn. Upon seeing the “monster”—blind, immobile with only one limb, suffering from congenital heart disease, and with no signs of mental capacity—approval was quickly given. This incident led to the development of a proper bureaucratic infrastructure capable of supporting and carrying out a child euthanasia program. The child euthanasia program laid the groundwork for the T4 adult euthanasia program, and both enabled the extermination of people with physical defects, genetic defects, and psychological abnormalities. The program was widely accepted by the medical community and strongly opposed by the public.^28^ Officially, the program would only be observed until 1941; unofficially it continued to the end of the war.

### Rationalization via the Hippocratic Oath

From a pseudo-scientific perspective that embraced social Darwinism and racial hygiene, the removal of any potential carrier of a “sick genetic line” was essential to the breeding of a genetically healthy Aryan nation.^30^ Hence, opposition to euthanasia by medical professionals waned in light of its legalization. One may well ask: but what about their Hippocratic oath? Wolfgang Schütz simplistically believes that the doctors ignored their oath for the sake of their careers.^31^^(pS281)^ However, German physicians, among others, had a very high regard for the Hippocratic oath, which at its heart enjoins the physician to “do no harm.”^29^,^32^ The answer is far more complex. In addition to the medical community buying into a so-called “science” of racial purity, and being legally free to pursue any means without compunction, the Nazi machine disseminated educational materials that raised up a new generation to have a complex interpretation of their duties, oath, and actions.^2^,^29^ The writings of Rudolf Ramm significantly impacted understanding of the Hippocratic oath under the Nazi regime. He was a general practitioner and politician who climbed the ranks of Nazi Party membership to become the Third Reich’s medical policy-maker. His book, “Medical Jurisprudence and Rules of the Medical Profession,” written in 1942, would provide guidance to the Third Reich’s future doctors, with scientific and moral justifications for their actions.^2^,^29^,^32^ Rather than a physician’s highest responsibility being the health and well-being of their patient unto death, their commitment was now directed toward a “racially pure folk [*Volk*]” and caring for only the healthy and hereditarily sound members of the population.^2^^(p32–3)^ Hence, murdering patients with disabilities or genetic diseases had no consequences since it was no longer considered an infliction of harm.

## EXAMPLES OF NAZIFIED DOCTORS AND NAZIFIED MEDICAL FACILITIES

The main question examined herein is: What motivated the German and Austrian doctors to actively participate in the implementation of the Third Reich’s ideals? Based on the above historical background, a number of rationales are possible, including: anti-Semitism and jealousy of the success of their colleagues, professional advancement, scientific ideology, love for their nation expressed via political ideology, self-preservation, or a complex mix of them all.

While a satisfactory answer may never be gained, a closer look at some well-known Nazi doctors could be enlightening. This article looks at the lives of four individuals from a few perspectives: their background before World War II, the possible professional benefits experienced due to the Medical Professional Elimination Program, their actions for the duration of the Third Reich, and their post-war lives. Doing so may clarify the forces that motivated them.

### Irmfried Eberl (1910–1948)

Born in Bregenz, Austria, Eberl was raised in a family that supported the pro-Aryan theories followed by the German nationalist movement in Austria.^33^ Eberl joined the Nazi Party in 1931, before the ascent of the Third Reich, while still a medical student. Eberl was not considered to be a particularly gifted student and had to repeat his final medical examination before graduating in 1935. In 1938, having moved to Berlin, he married a well-off influential woman who was a committed German national socialist.^34^

Although Eberl was not known to have been involved with the Medical Professional Elimination Program, he benefited from its aftereffects: with only limited training and experience, he became head of a prison that had been converted into a psychiatric institution—the Brandenburg Facility—in 1939. After euthanasia was progressively legalized, first for children and then adults between 1939 and 1940, Eberl oversaw the mass killings of 9,772 German patients labeled as “mentally ill.”^28^ That “success” led to his appointment of a similar facility in Bernburg with 8,601 victims.

Eberl’s next major advancement was as commander of the Treblinka extermination camp in April of 1942—the only medical doctor who served as a head of such a camp in the Third Reich. Knowing that a medical presence helped created a calm and trusting atmosphere, he was known to walk through the camp in a white coat, such that prisoners entered the gas chamber in an orderly manner.^34^ However, his poor administrative skills could not compensate for the efficiency of the killings. Despite murdering some 280,000 people within six short weeks—one of the highest killing rates during the Holocaust—Eberl failed to accommodate for the disposal of the dead and was quickly dismissed from his position.^33^,^34^

He eventually served as a physician on the Western front and was subsequently captured by the Allied Forces. Evading detection, Eberl was eventually released on July 6, 1945 and integrated back into society. However, during the Nuremberg Trials his name kept being mentioned by witnesses, leading to his arrest in January, 1948. Less than a month later, Eberl committed suicide.

The removal of members of the medical profession due to the Medical Professional Elimination Program clearly paved the way for ambitious men to achieve otherwise unattainable professional advancements. Eberl would seem to have been one such person. However, that ambition, seen through the eyes of his “achievements,” reveals a man whose medical ethics were driven by his own sense of power and control along with a full commitment to the Nazi ideology.

### Kurt Heissmeyer (1905–1967)

Born in Lamspringe, Germany on December 26, 1905, Kurt Heissmeyer was raised in a nationalistic medical family that envisioned restoration of the German empire. As a student, Heissmeyer was active in the German student’s union, *Arminia*, which strongly supported the nationalist agenda. After graduating from the medical school in Freiburg-im-Breisgau in 1932, he completed his internship in medicine. Although a mediocre student,^35^,^36^ he was subsequently employed at the Auguste-Viktoria Sanatorium in Hohenlychen in 1934, specialized in internal and lung diseases, including the treatment of tuberculosis—this thanks to his family connection as a nephew of the influential SS general, August Heissmeyer. After joining the Nazi Party in 1937, he was appointed chief lung physician at the sanatorium in 1938, and remained at Hohenlychen until 1945.

He wrote multiple publications between 1938 and 1943 that focused on racial hygiene, tuberculosis, and disease control—all with an anti-Semitic slant. For example, his 1943 paper on “Principles of Present and Future Problems of TB Sanatoria” argued that Jews, who were racially inferior, were less resistant to diseases such as tuberculosis.^37^ His contacts in high places paved the way for unparalleled horrific medical tuberculosis experimentation on mostly Jewish adults and children, whom he viewed as ideal experimental subjects due to their inherent weaknesses.^35^–^40^ Yet, he knew nothing about research and accepted the Nazi ideology on race as science.^35^ Hence, Heissmeyer’s “achievements” merit further scrutiny.

The state of tuberculosis research at the time in relation to Heissmeyer’s work has been thoroughly discussed elsewhere.^36^,^38^–^40^ Conflicting accounts credit his experiments in tuberculosis research to the encouragement of colleagues while drinking,^36^ or efforts for professional advancement to either receive a habilitation degree^9^,^39^ or a professorship.^40^ Among other topics, he wanted prove that a second peripheral skin infection with live tuberculosis bacteria would heal a primary central lung infection – a concept that had already been disproven in the literature. He specifically asked for and received permission to perform experiments on Jewish human subjects. Given that his research question and other assumptions had already been scientifically disproven in the literature,^35^–^40^ why was his work approved by the Nazi medical establishment, and why did he press ahead so vigorously to what was a foregone conclusion (i.e. failure)?

This question can be answered in part by noting that the medical establishment was in flux due to other actions being taken by the new government to control medical practice. Among them were mandatory indoctrination classes for younger physicians. In parallel, by the 1940s, tuberculosis was beginning to affect critical numbers of the German population.^9^^(p42)^ Hence, approval for his “research” was probably due to the Nazi ideology and the desire to blame the Jews for the increased tuberculosis scourge.

Heissmeyer’s human experiments began mid-1944 in the Neuengamme concentration camp, near Hamburg, and included infecting 60 Polish and Russian prisoners of war, and 10 Jewish girls and 10 Jewish boys between 5 and 12 years of age, with tuberculosis.^35^–^40^ He persisted in his research despite failed tests in all patients and had all of his patients murdered—the 20 children were “destroyed”^35^^(pviii)^ on the Führer’s last birthday, April 20, 1945.

After the war, Heissmeyer evaded detection and set up private practice as a tuberculosis specialist in Magdeburg, East Germany. He was eventually identified and, after a three-year trial, imprisoned in 1966, where he died a year later. During his trial, in answer to a judge’s questions, Heissmeyer stated that he did not initially perform tests on guinea pigs since he saw no difference “between Jews and guinea pigs.”^35^^(p114)^ The judge stated that of all the atrocities in the war, this must be the most callous crime.^38^

Heissmeyer was a young physician in pursuit of professional recognition, advancement, and social prestige—all of which became more available to him once the supposedly inferior Jewish “competition” had been removed from the picture. Furthermore, his anti-Semitism and political leanings aligned with the Nazi regime. The Medical Professional Elimination Program was fortuitous for Heissmeyer, facilitating his professional advancement, highly questionable “scientific” efforts, and ideology.

### Ernst Ferdinand Sauerbruch (1875–1951)

Born on July 3, 1975 in Wuppertal-Barmen, Germany, Professor Ernst Ferdinand Sauerbruch is highly respected within modern medicine as the father of cardiopulmonary surgery. However, he leaves an ambiguous legacy worthy of further consideration. His medical achievements are undisputed (Table 1) and underestimated. His surgical techniques continue to save countless lives.^42^ His preeminence as a researcher and surgeon was already undisputed in the 1930s. He needed nothing from the Nazi Party to advance his career. Hence, an examination of the words and actions of Sauerbruch before, during, and after the Third Reich is revealing.^43^,^44^

**Table 1:** Major Medical Innovations of Ernst Ferdinand Sauerbruch.^41^

Year	Innovation	Description
1904	Sauerbruch chamber	A negative-pressure chamber for thoracic surgery, enabling safer heart and lung operations
1907	Sauerbruch grip	A methodology for achieving hemostasis during cardiac surgery
1915	Prosthetic limbs	Advanced prosthetic limbs for war veterans that, for the first time, enabled improved movement
1931	Successful surgery of cardiac aneurysm	An accidental discovery involving rapid application of silk sutures in blind knots to isolate the aneurysm
Throughout his career from 1904 onward	Additional thoracic surgery innovations	Pioneered techniques for thoracoabdominal injuries, lung resection, esophageal cancer, pericarditis, tuberculosis treatment, cardiopulmonary surgery; combined, made him the pioneer of thoracic surgery

Concomitant with his fame and utter dedication to medicine and research, historians note Sauerbruch’s drive to succeed, his organizational capabilities, arrogance, and pride, and his ferocious anger, once ignited.^41^,^43^–^45^ Sauerbruch was an authoritarian leader, enjoyed the adulation of his peers and patients, and was not averse to leveraging his professional relationships with highly placed colleagues in the Third Reich for both professional and personal reasons.^43^ These relationships and his professional achievements led to professional advancement and preeminent prestige and respect within the German medical community, the Third Reich, and worldwide. Sauerbruch may have held an over-inflated perspective of his own importance, but the Nazis recognized his importance and leveraged it to their own purposes.^45^ Although his name was on the original list of physicians considered for prosecution at the Nuremberg trials and subsequently removed, he also testified at the trials in defense of other Nazi physicians.^46^

Rather than providing a full biographical survey of his life, which has been documented in multiple publications,^41^–^45^ the authors present data regarding Sauerbruch’s activities, from the pre- to post-war years, in an attempt to better understand his ambiguity with regard to the Third Reich and its leaders (Table 2).

**Table 2:** Documented Statements and Activities Reflecting the Support or Resistance of Ernst Ferdinand Sauerbruch to the Nazi Regime.^*^

Date	Documented Activity of Professor Sauerbruch
1918–1927	Chief of Surgery, University of Munich; becomes acquainted with Karl Gebhardt and Karl Brandt who trained under his leadership^46^
1918–1919	Makes numerous public statements calling for “German unity, to regain the place that the German fatherland deserves after all the humiliations”^43^^(p203)^
1920	Introduced to the future Führer by Dietrich Eckart (first editor-in-chief of *Volkischer Beobachter* after coming under NSDAP) in Munich, asked to spread anti-Semitic propaganda among students^43^
1923: November 11	States that although the future Führer had good qualities and that he believed in him, he would always resist him politically^43^
1923: January 20	Mentions a recent conversation with the future Führer in a letter^43^
1923: September/October	Meets with the future Führer in private clinic^43^
1923: November 8–9	Treats the future Fuhrer’s injured left shoulder after the failed Beer Hall Putsch (attempt to overthrow the current government); fully informed on the course and outcome of the putsch^43^
1923: November 10	Sends an assistant to the future Führer in Uffing^43^
1927–1949	Director of Surgery, Charité in Berlin
1933: March–November	Oversees the dismissal of 13 Jewish physicians in accordance with the Professional Elimination^47^
1933: September	Publishes an open letter to the medical professionals of the world; regarding his cooperation, states that he is “seriously concerned about the initial side effects” … [but that] “hard and difficult interventions that accompany every revolutionary act” should not obscure the “‘greatness’ of this (‘our’) revolution”^43^^(p211–12)^
1933	Publicly supports the National Socialist rise to power in multiple speeches^43^,^44^
1934: March	Dismisses a Jewish surgical associate for no known reason^47^
1934: December	Joins the main committee after dissolution of the Emergency Association of German Science^43^
1935 (throughout)	Dismisses 11 Jewish associate professors and doctors of surgery following passage of the Nuremberg Laws^47^
1937	Becomes a member of the Reich Research Council; from 1941 onward, one of his tasks is signing documents approving medical research on humans in concentration camps or asylums^44^
1937: January 30	Receives the German National Prize for Art and Science
1937: September 18	Publicly expresses gratitude for the award from the Führer^43^
1939: March 5–7	Main speaker at the Reich Conference on Public Health and Genetic Poisons^43^
1942	Becomes a General Physician Inspector of the German Military Health Services, a member of the Academy of Military Physicians^42^,^44^
1942: End of May	The Führer and Himmler urge K. Gebhardt to call in Sauerbruch to treat Heydrich (did not happen)^43^
1943: February	Visits Mussolini who was ill^43^
1943: May	Attends a meeting of the Berlin Military Medical Academy^43^
1943: Late Autumn	Receives the Knight’s Cross to the War Merit Cross at a meeting of the German Society for Surgery
1943: November 9	Thanks a Reichsleiter of the NSDAP for congratulations on the award^43^
1943: December	Goebbels’s Propaganda Ministry plan a “non-political anti-Bolshevik united front” involving Sauerbruch (the event was never held)^43^

* Unreferenced items appear in multiple publications discussing his life

Despite his relationships with Nazi leaders (including treating the future Führer’s shoulder injury in Munich soon after World War I), Sauerbruch never joined the Nazi Party. Furthermore, he was known to publicly disagree with certain Nazi policies, but, unlike other physicians, he was unscathed by the experience.^43^–^45^ He opposed the T4 euthanasia program (though only for Germans), yet as a member of the Reich Research Council he approved medical research documents enabling research on humans in the concentration camps.^44^ He enabled a Jewish colleague to escape Germany, yet approved the dismissal of multiple Jewish colleagues in compliance with the Medical Professional Elimination Program (Table 3). He was friends with collaborators in the 1944 plot to assassinate the Führer, and his own son was arrested as an accomplice in the plot; yet he fearlessly used Nazi connections to obtain his son’s release.^43^,^46^

**Table 3:** Medical Staff Eliminated under Sauerbruch’s Leadership at the Medical Faculty, Charité in Berlin.^47^

Name	Professional Information	Birth-Death (Years)	Legal Clause for Dismissal^*^	Dismissal Date	Outcome
**Aschheim**, Selmar	Gynecology and Biology	1878–1965	§ 4 RBG - Deprives Jews of citizenship, required dismissal from work	1935	Emigrated to France
**Bergmann**, Ernst W.	Orthopedic Surgery	1896–1977	§ 4 RBG - Deprives Jews of citizenship, required dismissal from work	1935	USA
**Borchardt**, Moritz^†^	Associate Professor of Surgery	1868–1948	§ 3 - Non-Aryan descent	Mar 4, 1933	Emigrated to Argentina
**Casper**, Leopold	Associate Professor of Urology	1859–1959	§ 3 - Non-Aryan descent	Sep 14, 1933	Emigrated to USA
**Freund**, Richard	Associate Professor of Gynecology and Obstetrics	1878–1942	§ 3 - Non-Aryan descent	Sep 14, 1933	Suicide after deportations began
**Gerlach**, Walter	General Surgery	Unknown	§ 3 - Non-Aryan descent	Unknown	Unknown
**Heydemann**, Hans	Orthopedic Clinic Assistant	1898-?	Unknown	Unknown	Unknown
**Heymann**, Emil^‡^	Chief Surgeon in Surgical Department	1878–1936	§ 4 RBG - Deprives Jews of citizenship, required dismissal from work; noted as furloughed in 1935 as “non-Aryan”	1935	Suicide
**Israel**, Arthur	Associate Professor of Surgery	1883–1969	§ 4 RBG - deprives Jews of citizenship, required dismissal from work	1935	Escaped to USA; returned to Munich in 1969
**Israel**, Wilhelm James	Surgery/Urology	1881–1959	§ 4 RBG - Deprives Jews of citizenship, required dismissal from work	1935	Emigrated to UK
**Joseph**, Eugen	Professor of Urology and Surgery, Head, Department of Urological Surgery	1879–1933	§ 3 - Non-Aryan descent	Sep 4, 1933	Suicide
**Josephs**	Doctor in First Surgical Clinic	Unknown	§ 3 - Non-Aryan descent (uncertain)	Unknown	Unknown
**Kisch**, Eugen	Associate Professor of Surgery	1885–1969	§ 3 - Non-Aryan descent	Sep 4, 1933	Emigrated to Brazil
**Klein**, Paul	“Privatdozent” [lecturer] in Gynecology and Obstetrics	1892-?	Teaching license revoked	1933	Unknown
**Landau**, Hans	Associate Professor of Surgery	1892–1935	§ 3 - Non-Aryan descent	Sep 9, 1933	Emigrated to UK
**Lichtenberg**, Alexander von	Associate Professor Surgery/Urology		§ 4 RBG - Deprives Jews of citizenship, required dismissal from work	1935	Emigrated to Mexico
**Liepmann**, Wilhelm	Associate Professor of Gynecology and Obstetrics	1878–1939	§ 6 - Retired for no known reason	May 3, 1933	Emigrated to Turkey
**Mannheim**, Hans	Assistant, Surgical Clinic	1900–1972	§ 3 - Non-Aryan descent, initially on leave from May 15, 1933, but dismissed on Jun 29, 1933	Jun 29, 1933	Emigrated to UK and USA
**Marcus**, Max	Surgery	1892–1983	§ 6 - Retired for no known reason	Mar 14, 1934	Emigrated to Palestina (pre- Israel) via Lebanon
**Meyer**, Robert O.	Honorary Professor of Gynecology and Histology	1864–1947	Retired in 1935	1935	Emigrated to USA
**Nissen**, Rudolf	Associate Professor of Surgery	1895–1982	§ 18 RHO^¶^	Jul 15, 1935	Emigrated to Turkey/USA/Switzerland
**Picard**, Hugo	Associate Professor of Surgery	1888-?	§ 3 - Non-Aryan descent	Jul 15, 1945	Emigrated to Egypt
**Pribram**, Bruno Oskar	Associate Professor of Surgery	1887-?	§ 3 - Non-Aryan descent	Nov 24, 1933	Remained in Berlin until February 1938; final destination unknown
**Salomon**, Albert	Associate Professor of Surgery	1883–1976	§ 3 - Non-Aryan descent	Sep 8, 1933	Emigrated to Holland
**Sauer**, Hans von	Scientific Assistant in Surgical Clinic	1903-?	§ 3 - Non-Aryan descent	1933	Emigrated to USA
**Schück-Breslauer**, Franz	Associate Professor of Surgery	1888–1958	Teaching license revoked in 1935	1935	Emigrated to USA; requested to return to Berlin in 1957
**Strassman**, Paul F.^**^	Associate Professor of Obstetrics and Gynecology	1866–1938	§ 4 RBG - Deprives Jews of citizenship, required dismissal from work	Oct 19, 1935	Emigrated to Switzerland
**Strassmann**, Erwin O.	“Privatdozent” [lecturer] in Gynecology and Obstetrics	1895–1972	§ 4 RBG - Deprives Jews of citizenship, required dismissal from work	Oct 19, 1935	Emigrated to USA
**Zondek**, Bernhard	Associate Professor of Obstetrics and Gynecology	1891–1966 (New York)	§ 3 - Non-Aryan descent	Sep 5, 1933	Emigrated to Palestina (pre-Israel) via Sweden

Professor Sauerbruch directed the surgical department from 1928 to 1949. The majority of dismissals were in 1933 under clause § 3 of The Law for the Restoration of the Professional Civil Service,^48^ which officially initiated the Medical Professional Elimination Program. Likewise, dismissals under one of the Nuremberg Laws occurred soon after they became official.

* The reasons for dismissal were included in the original list of employees dismissed based on clause number and year of the 1933 Professional Civil Service Act,^48^ unless marked with the letters “RBG” (*Reichsbürgergesetz*, i.e. the 1935 Reich Citizenship Law [one of the Nuremberg Laws])^49^ or “RHO” (*Reichsheimstättengesetz*, the Reich Homestead Law).^50^ Each clause of these laws carried with it important nuances. Hence, this table provides the clause number as provided (e.g. § 3 or § 4 RBG, etc.), followed by a brief description of the nuanced reason for dismissal. The reader is referred to the references for more details about the law and clause enacted.

† Borchardt stands out as the only doctor from this group who was dismissed on March 4, 1933, *before* the Civil Service Act officially became law, yet he is marked as having been released based on it.

‡ Although Heymann is recorded as being a Protestant, he was classified as a non-Aryan and suspended from his work. The terminology used regarding his “dismissal” was actually “furloughed.”

¶ Clause 18 of the RHO relates to the transfer of funds to departments based on the established budget and circumstances when an audit is performed. The cited reason for dismissal is interesting since Nissen trained under Professor Sauerbruch at the University of Berlin and transferred with him to the Charité. Nissen’s dismissal was delayed since he had served in World War I and was exempt from dismissal. According to Berry, Sauerbruch was instrumental in Nissen’s escape from Germany.^51^

** Paul F. Strassman is noted to have been a Protestant. It is possible that he had a Jewish spouse or someone Jewish in his lineage.

A careful examination of Table 2 reveals a man who supported the Nazi regime, most likely due to his patriotic desire to see Germany regain its former status. He viewed the negative aspects of the regime as a necessity that would eventually pass once the revolution had achieved its goals (Germany’s restoration to greatness).^44^ The boldness with which Sauerbruch chose to refute certain Nazi policies while ignoring others reflects both a pragmatic approach toward the regime as well as confidence in his own position and personal safety.^43^,^44^

After the war he faced denazification, despite protests from his professional colleagues. Descriptions of the trial would seem like a comedy of errors if the subject matter were not so serious.^43^,^45^ Sauerbruch’s defense was one of belligerent anger aimed at undermining the legitimacy of the trial.^45^,^46^

Following the death of a patient in 1949, he was forced to retire. That death marked the culmination of several years of increasing morbidity and mortality noted in his patients, most likely due to Sauerbruch’s degenerating mental condition, a condition that he denied until his death. Sauerbruch’s denial led to the disability and even death of several patients who implicitly trusted him and sought out his care.^44^,^45^

Medical history may recognize Sauerbruch’s greatness as the Father of Thoracic Surgery, but his life is a cautionary tale of an authoritarian, egocentric, and proud man who had the influence, both personally and professionally, to play both sides—but at what cost? Ultimately, his signature as head of the Reich Research Council facilitated medical experimentation at the Natzweiler and Auschwitz concentration camps, including some of Mengele’s medical research experiments.^43^,^52^,^53^^(p76)^

### Lorenz Böhler (1885–1973)

Lorenz Böhler was born in Wolfurt near Bregenz in upper Austria on January 15, 1885. His interest in medicine began at an early age. Despite family problems that led to his failure in school, he moved to Vienna to pursue medicine and became a Doctor of Medicine in 1911. A fervent patriot with a great interest in surgery, he volunteered during World War I, was assigned to a military hospital, and worked there as a surgeon from 1914 to 1916, becoming the leading surgeon in 1916.^54^ That experience led to an exceptional career focused on traumatic injuries.^54^,^55^ In addition to excelling in bone surgery, he developed methodologies for protecting injured limbs, suspension and immobilization, muscle strengthening, gradual activation, and rehabilitation, and his standardized conservative method for treating bone fractures would spread worldwide.^55^ Böhler eventually became known as the Father of Accident Surgery.

Following World War I, despite much resistance, he established an accident hospital in 1925.^56^ The hospital became recognized for its excellent patient care, superb organization, successful treatment of long bone fractures, and realization of huge savings on the part of insurance companies. In 1929 he published the first of more than 13 editions of his seminal work, *Die Technik der Knochenbruchbehandlung* (The Technique of Fracture Treatment). His success led to an appointment as lecturer in surgery at the University of Vienna in 1930; he became a full professor of accident surgery in 1936. Böhler’s renown attracted patients from around the world.^57^ He did not hesitate to use innovative ideas that could potentially help injured patients return to normal function. Of particular relevance was his support of the Küntscher nail for femoral fractures, which was initially rejected by German surgeons. His advocacy of this technique led to its eventual use mostly in Germany and Austria in the 1940s.^54^,^55^ By 1948 he had published 11 editions in German of his volume, *Medullary Nailing of Küntscher*.^56^

Böhler, a believer in highly organized processes and documentation,^55^ was known as a liberal nationalist who was impressed by the organizational structure of the Nazi system.^56^ Despite conflicting reports (Böhler’s testimony versus Nazi documents), the most reliable records reveal that Böhler joined the National Socialist German Workers’ Party (NSDAP, i.e. the Nazi Party) on February 12, 1938 (before the *Anschluss*) and received his membership number, 6,361,999, the next day.^59^ Membership in the NSDAP prior to the *Anschluss* was considered illegal after 1945. These and other activities of Böhler have been detailed by Nemec and are summarized in Table 4.^59^ Of interest, he became a supporting member of the SS but was not considered a regular member. Rather, Böhler would have given financial contributions, received the SS journal *Das schwarze Korps* regularly, and was understood to be a member of the SS community, but without rank in the SS hierarchy and with no specific orders, responsibilities, or tasks (private communication from Professor Michael Hubenstorf, May 15, 2024). Böhler also received three prestigious awards from Nazi Germany between 1941 and 1945 (Table 5).^59^ These awards reflect Böhler’s affiliation with the Nazi regime and his support of the Nazi ideology.

**Table 4:** Documented Memberships and Activities Reflecting the Support or Resistance of Lorenz Böhler to the Nazi Regime.

Date	Documented Activity of Professor Lorenz Böhler
1934, March 4	Becomes a member of the *Vaterländische Front* (a right-wing conservative nationalist Austrian organization that banned political opponents including communists, social democrats, and Austrian Nazis; disbanded by the Nazi Party immediately after the *Anschluss*)
1938	Joins the National Socialist People’s Welfare organization (NSV)
1938	Joins the Reich Air Protection League (RLB)
1938, February 12	Joins the NSDAP (retrospectively considered illegal from 1945; implies voluntary membership prior to the *Anschluss*)
1938, February 13	Receives membership number 6,361,999.65 in the NSDAP^57^
1939, June 18	Becomes supporting member of the SS, membership number 1,415,799; SS- *Abzeichen-Nr*. 18,403.74^57^
1940, March 19	Joins the National Socialist German Doctors’ League (NSDÄB), an organization that sought to align the medical profession with Nazi ideology and policies
1940–1945	Serves in the Wehrmacht as: Consulting surgeon for Army Group 5 Oberfeldarzt (Medical Staff Officer), Vienna Rudolfspital in Reserve Hospital XIa^*^ Oberfeldführer (Senior Field Leader), Landesstelle XVII, Medical Faculty’s Deanery
1943, September 20	Leo Eigenthaler provides a sworn statement supporting Böhler’s late (and therefore acceptable) NSDAP membership
1943	Blacklisted by the SS because of his suspect political stances, and denied paper to reprint his books (testimony of Böhler)
1944, January 26	Gau personnel office issues letter confirming Böhler’s NSDAP membership date
1945, April	Decommissioned from the Linz Lazarett (Reserve Hospital)^60^ (XI-a)^*^ (testimony of Böhler)
1945, September 20	Date of Böhler’s sworn statement that he joined the NSDAP under duress only after the *Anschluss* (Spring, 1938)
1945	Dismissed from the University of Vienna
1947	Cleared of all charges due to advocacy of Karl Renner and reinstated at University of Vienna

All data taken from Nemec^59^ except where otherwise indicated.

Gau, Gaupersonalamts (an NSDAP office responsible for managing personnel matters within a specific regional division); NSDAP, National Socialist German Worker’s Party.

* The XI-a or XIa designations indicate that both facilities were part of the same medical unit network. Hence, these entries imply that Böhler may have been stationed in Vienna, but, for reasons unknown, he was deployed to Linz, from where he was decommissioned.

**Table 5:** Awards Received by Professor Lorenz Böhler between 1941 and 1945.^59^

Date	Award	Description
1941	Eisernes Kreuz, II. Klasse (Iron Cross Second Class)	A military decoration awarded by Nazi Germany for acts of bravery or leadership in combat
1942	Kriegsverdienstkreuz 1. Klasse mit Schwertern (War Merit Cross First Class with Swords)	Established by the Führer in 1939, given for meritorious service connected with the war effort
1945	Prinz-Eugen-Medaille (Prince Eugen Medal)	Associated with the 7th SS Volunteer Mountain Division “Prinz Eugen,” a unit of the Waffen-SS (armed wing of the Nazi Party)

Tables 4 and 5 present an impressive résumé. How could Böhler claim, despite the support of prestigious people such as Leo Eigenthaler and Karl Renner, president of the Austrian Republic, that he had no special activities or connection with the Nazi Party in an official form submitted to the University of Vienna in 1946?^60^ Just the conflicting NSDAP membership dates should raise eyebrows, in light of Böhler’s known admiration for processes, procedures, and accurate documentation.^54^,^56^

Little is known of Böhler’s actual activities, and there is no direct evidence of his complicity with the Nazi regime. However, a 1939 political assessment of Böhler, prior to his being accepted as an officer in the *Wehrmacht*, noted him as being nationalistic and anti-Semitic.^59^ Being placed on the SS blacklist for his political statements could have stood in his defense, except this was his own testimony, uncorroborated by external documentation.^59^,^61^

A brief look at the role of the Medical School of the University of Vienna during the Third Reich may be enlightening, since Böhler was one of its most famous faculty members.

#### Böhler and the University of Vienna

Firstly, even before the *Anschluss*, the University of Vienna is historically recognized as having been one of the most aggressive in its anti-Semitic stance. The university was home to a secret group (the “*Bärenhöhle*” [Bear Cave]) of anti-Semitic professors. Their influence prevented numerous Jewish professionals from completing their habilitation degree and working at the university.^62^ Two days after Austria’s incorporation, on March 15, 1938 the university appointed Professor Eduard Pernkopf, a strong Third Reich supporter, as dean of the university’s faculty of medicine. His zeal for the Medical Professional Elimination Program was evident: Pernkopf oversaw the dismissal of over 150 non-Aryan faculty members (77.5% of the medical faculty^16^) and required all remaining professors and lecturers to give an oath to the Führer.^10^ The University apparently supported these actions since, in total, 2,700 faculty members were dismissed, most of whom were classified as “Jewish.”^63^

It is assumed that Böhler gave the required oath since he remained in the faculty. Given Böhler’s memberships (Table 4) and critical role as a trauma surgeon and lecturer in both the university and the community, he must have been fully aware of the university’s anti-Semitism and support of the Third Reich racial hygiene laws. Hence, his signature on a letter addressed to the American Medical Association (AMA) is of interest.^10^,^64^ A recommendation had been made to move the Vienna branch to London.^64^,^65^ One reason given included the large number of dismissals from the university for political and racial reasons. Several of the remaining doctors, all AMA lecturers, would have lost substantial income if the Vienna branch moved, including Böhler, whose lectures were in high demand.^65^^(p501)^ He and other colleagues signed and submitted a letter stating:

“The undersigned know of not one case of prosecution of a professor for his racial or religious adherence. … The truth is that Jews are no longer allowed to teach non-Jews. … By the removal of certain influences, a trend of charlatanism … was eliminated.”^10^^(p791)^

The Vienna branch remained in place throughout the war, but largely inactive until it ended.^65^^(p503–4)^

#### The Motivating Why for Böhler

Böhler was a preeminent surgeon whose work remains relevant to this day. Like Sauerbruch, his prestige and accomplishments were well-established before the Nazi Party came into power. As a member of the University of Vienna faculty, it is not surprising that Böhler faced a possible trial in 1946. However, the intervention of his friend Karl Renner, president of Austria, led to his amnesty in 1947. Böhler maintained his worldwide reputation and spent his final years in retirement traveling and teaching.

While he played no role *per se* in the Medical Professional Elimination Program, Böhler could not have been unaware that 77.5% of his colleagues had suddenly disappeared because of their Jewish ancestry. Furthermore, there is undisputed testimony from the *Betriebsrat* (the work council representing employees) of the Accident Hospital in Vienna that Jewish physicians were not accepted to work there (private communication from Professor Michael Hubenstorf, May 15, 2024).

Böhler insisted that he had been forced to join the Nazi Party, but, if this is true, why the discrepancies between verbal testimonies and the Nazi documentation?^59^,^61^ No explanations have been offered for any of his other memberships and activities. Why did he sign the letter to the AMA? Was it *only* to maintain his income and prestigious position? What did he *do* to merit receiving three prestigious awards from the Nazis—one from the Führer himself (Table 5)? These awards alone merit further examination. As Nemec and others have concluded, Böhler’s participation in the Nazi Party and possible culpability in racial discrimination remain to be investigated.^59^,^66^

### Medical Institutions and Their Practitioners

The above examples are not isolated ones. Large numbers of medical professionals worked together to help the Nazi machine fulfill its ideology. Hence medical institutions were also considered Nazified. In the discussion of Böhler, only a few examples of the University of Vienna’s cooperation with the Third Reich were mentioned. The following testimonies reveal that more institutions were involved, along with their personnel.

#### Account of Holocaust Survivor, Dr Albert Haas

Dr Albert Haas was a physician with an undisclosed Jewish background who was arrested for political activities. Classified as an Aryan with prisoner no. 1,762,222, he served as the chief physician of the Gusen II subcamp at Mauthausen.^27^ Haas provides interesting details about the medical experiments performed on the Gusen prisoners by respected German pharmaceutical companies and medical institutions. Of particular interest is his detailed description of experiments performed there. Haas writes:

… a famed Viennese orthopedic clinic was interested in improving the existing technique for repair of a fractured hip. They conducted a critical research project at Gusen II. The clinic’s medical researchers smashed prisoners’ hip joints in order to try out a variety of surgical techniques and artificial joints.^67^^(p334)^

Haas never identified the “famed Viennese orthopedic clinic.” However, based on the known facilities of repute at that time, there are only a few possibilities: The University of Vienna Medical School, Vienna General Hospital, the Orthopedic Hospital of Vienna-Speising, The Lorenz Böhler Trauma Hospital, and the Rudolfspital.

It is unclear which institute was involved. However, of interest, the Trauma Hospital in Vienna remains conflicted regarding its founder’s possible past Nazi allegiance and service,^65^ and the University of Vienna was known to be a participant in Nazi research facilitated by receiving the bodies of executed political prisoners for autopsy.^68^,^69^

#### Account of a Patient from Linz

**Figure 1:**
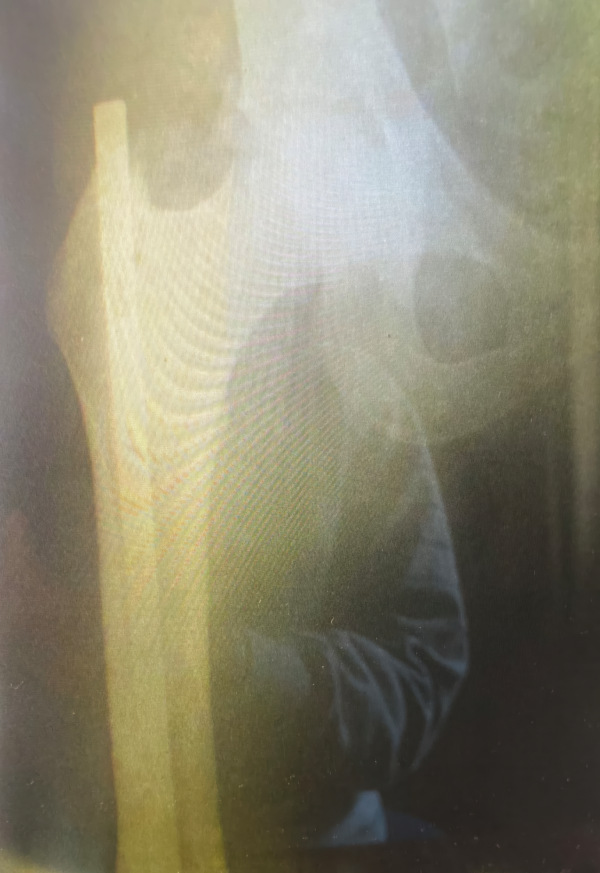
X-ray of Ukrainian Claimant Submitted to German Authorities for Reparations. The femur (thigh bone) with the Küntscher intramedullary nail is seen; there are no signs of callus or a healed fracture line, despite the poor quality of the photograph. Photo provided courtesy of M. Dubyk.

An interesting orthopedic case was brought to the attention of coauthor GMW a few years ago via a Ukrainian publication.^70^ The case was presented at an international conference in London and a copy of the X-ray provided (to GMW by email) (Figure 1), along with additional information. The patient had asked for compensation from Germany for an injury incurred at Mauthausen, after which he had undergone surgery in the Linz hospital. The author wrote:

In Linz, after the bombing, the applicant underwent an experimental operation to insert a metal rod into the bone marrow canal of the thigh (Küntscher nail), although the leg injury was not documented in the hospital records.^70^^(p43)^

However, follow-up with Linz hospital was not possible since their records for that time period, as stated, had been lost.

It is known that the Germans used the Küntscher nail on prisoners of war^55^ and that Böhler was instrumental in training others in its use.^58^,^71^ Perhaps he facilitated use of the Küntscher nail during his deployment in Linz, from which he was demobilized in 1945.^60^ However, once he taught the procedure to others, he could not be responsible for its use—or abuse—in Linz or elsewhere.

## DISCUSSION AND CONCLUSION

The Medical Professional Elimination Program was clearly a political tool in the hands of the Nazis to achieve their goals. However, it was facilitated by an entrenched ideology that was already taking hold well before the Nazis appeared. It has been stated herein that the Medical Professional Elimination Program facilitated all that eventually happened in the Holocaust. Is this an overstatement? The authors think not. Firstly, there seems to have been a core commitment to their respective nations, particularly in light of the first world war; others were genuinely committed to Germanic social Darwinism. Secondly, most Aryan physicians did indeed experience academic and financial gain once their non-Aryan colleagues (and they were many) were gone.^32^ While much has been written regarding the redefining of the Hippocratic oath by the Nazi doctors,^2^,^33^ the end result was that they ultimately did renounce their oath and did much harm.

The specific motivations of the illustrative physicians presented above are clearly different; yet whether they consisted of silence while their colleagues were banished, accepting membership in a medical organization established to promulgate the Nazi ideology, or becoming a perpetrator of death in the Nazi camps—all such actions should be incompatible with a physician’s medical oath and obligations. This presents a warning for all medical professionals: should the changing norms and theories of science, political affiliation, or society define physicians’ obligations to their patients? There is much to be said for the Judeo-Christian ethical respect for life. Indeed, this ethic was strongly renounced by the Nazis.^2^^(p33)^

None of the individuals discussed adhered to the Hippocratic oath and its commitments. Eberl ignored the rule to respect human life. Heissmeyer redefined the rule “to do no harm” since he equated Jewish lives with those of animals.^36^ Both were clearly motivated by a Nazified scientific ideology. But what were the motivations of Sauerbruch and Böhler? Perhaps many Austrian physicians supported the Nazis because of their careers,^35^ but that was clearly not the case for either man. Both were already known worldwide, well off financially, and in high demand. Sauerbruch considered himself without obligation to both colleagues and patients alike and signed life-destroying authorizations without consideration of the consequences. Böhler lived behind a veil of consummate professionalism while willingly joining organizations that actively supported the Nazi ideology. The organizations within which these individuals worked, and others which were mentioned in passing, were manned by professionals who were equally complicit.

A view that considers the Nazi physicians as victims of their circumstances negates the element of free choice and the ultimate responsibility physicians have toward their patients. This was the dilemma faced by judges and prosecutors alike during the Nuremberg trials. Many of the doctors interviewed were absolutely convinced of the ethical and moral right of their actions.^33^,^46^ This and other complex issues took precedence over determining whom to prosecute. Of the hundreds of physicians needed to power the machine of Nazified medicine, only 20 physicians were actually tried in Nuremberg, along with an additional three SS administrators.^46^^(p6)^

In 2012, the German Medical Association officially apologized and asked forgiveness for the crimes committed by their predecessors during World War II.^72^ However, they did not accept liability, their apology made no mention of the Holocaust,^10^ and it did not come from any of the perpetrators.^73^ In 1998 the University of Vienna issued an apology for its role in dismissing 173 professors and consultants.^17^ However, to the best of the authors’ knowledge, such an apology was never officially issued by the Republic of Austria or the Austrian Medical Chamber.

If given the gift of hindsight and alive today, would the original perpetrators finally have a sense of shame and apologize, or would they still rationalize their actions as having been done for the good of humanity’s genetic pool? Would they continue to redefine the Hippocratic oath, or would they feel some degree of remorse? When the German and Austrian medical professionals so eagerly dismissed their Jewish colleagues, was it only a helpless acceptance for the sake of survival? Could it have been a complex mix of envy, anger, and genuine hatred of the other? Were they absolutely convinced of the necessity for “racial hygiene” for the good of Aryan humanity? Or were they simply trying to navigate a moral climate and evolving society opposed to all that they had considered good?

History recounts an ideological betrayal of medical ethics by the Third Reich. However, this article has shown that, both ideologically and actively, the betrayal already existed in Germany and Austria. With the addition of Aryan racial science and the humiliation of Germany and the Austro-Hungarian Empire following World War I, that ideology further developed and was liberated from all legal constraints during the Third Reich. It intensified, expanded, and became lethal.

The non-obligatory abrogation of medical ethics by a large proportion of the medical profession during the Third Reich subjugated medical ethics to the rules of the country. Medicine became a voluntary instrument in the hands of the state, with catastrophic results—the devastating ideological betrayal of the noble profession of medicine.

This was the conclusion of the Lancet Commission on Medicine, Nazism, and the Holocaust:

The central insight from the history of medicine during Nazism and the Holocaust is that the atrocities that health professionals committed during the Nazi reign and the Holocaust represent, to a large degree, the outcome of corrupt moral agency in the face of potential dangers that are inherent to modern, scientific medicine as it emerged in the 19th century.^74^

In conclusion, the Medical Professional Elimination Program was the result of a pre-existent ideology, expressed well before World War I. It gradually spread throughout Germany and Austria during the interwar period and exploded during World War II, when Medicine lost its humanity and descended into NIGHT.

## Abbreviations

AMA: American Medical Association.
